# Bromine and Its Compounds

**Published:** 1866-12

**Authors:** J. H. Hollister

**Affiliations:** Prof. Chicago Medical College, Chicago


### ARTICLE XLIII.

### BROMINE AND ITS COMPOUNDS.

By J. H. HOLLISTER, M.D., and Prof. Chicago Med. College, Chicago.

Read to the Illinois State Medical Society, June, 1866.

At our last annual meeting, I proposed to prepare for consideration, an article upon "Bromine and its Compounds, as Curative Agents;" for, though at that time considerable interest had been excited with reference to its use, especially in our military hospitals, yet but little had been written upon the subject, and researches with reference to its literature were extremely unsatisfactory. *Since* then, however, the journals, almost without exception, have made abundant reference to it, and much that I then intended to express has been already anticipated, rendering it almost superfluous that I should complete the article as I had intended. Still, rather than fail of discharging the duty of preparing a report as ordered, as, too often, has been the case with our standing and special committees, I shall venture to present views and facts with which you may already be familiar, with the hope of eliciting additional knowledge from those who have had more abundant facilities for its trial and knowledge of its virtues. I deem a just estimate of its curative powers very essential to the interests of humanity: at the same time, I should consider its over estimate an equal misfortune, as, from being to sanguine of its power, disappointment might even deny for it the value which it may justly claim. Now, that the attention of the profession is so fully turned to it, I believe that men of acknowledged ability will soon assign for it a just place and proper estimate, as a remedial agent, and that it will render to us a much more essential service in the healing art than it has hitherto done.

I. Its natural history, in brief, is this:—Bromine was first discovered by BALARD, in 1826. He obtained it from that portion of sea water from which common salt had been crystalized, and it was first named by him "*muride*," from "*muria*," *brine*. When GAY LUSSAC took it in hand for examination, he conceived it to be the sum total and aggregate of all villainous stinks, and persuaded BALARD to call it "*brome*," or bromine, from the Greek "*bromos*," *the stink*. Neither of these men being familiar with the Chicago River, they deemed the bantling justly named.

So strong are its affinities, that it is never found in a free state, but, in various combinations, may be derived from both the mineral and organic kingdoms. In the organic world, it is obtained from many of the marine plants of the Mediterranean, in the stony concretions of sea sponge, and, especially is it to be noted, as one of the constituent elements of "*cod liver oil.*" Its inorganic combinations are too numerous to mention. In the waters of the Mediterranean, the Baltic, and the Dead Sea, and in very many of the salt springs of the old and new world, it is found in greater or less abundance. The German springs of Kreusnach are especially rich in it, and from this source it was formerly mainly obtained. More recently, it has been found in the salt springs of Salina; and in still greater abundance, so as now to mainly furnish the market, from the salt springs in Pennsylvania. Its main combinations in these waters are with calcium and magnesium. Of the manner of its preparation, I shall not speak.

II. *Physical Properties.*—Bromine, in its pure state, is an exceedingly volatile liquid; of a dark red color; pungent odor; acrid taste; boils at a temperature of 117° Fahrenheit, and solidifies at 4°; is not combustible; does not support combustion; immediately extinguishes a taper thrust into its vapor; is most soluble in ether; a powerful bleaching agent; colors the skin yellow, when in contact, as does nitric acid; and has a specific gravity nearly three times that of water.

III. *Forms of Administration.*—It has been highly recommended by Dr. M. GOLDSMITH and his associates, in the form of solutions of various strength, from 20 to 40 drops of bromine to the ounce of alcohol or ether; and still later, they advise its external application in its full strength. Internally, it is most frequently administered in the forms of bromides of potash and ammonia. Its vapor diffused through the air is, also, one form of its use.

IV. *Physiological Effects.*—The very concurrent testimony of a number of observers is to the effect, that bromine has a *decided sedative influence* upon the *cerebro-spinal nervous centres*, and that, as a result of such impression, its free use is followed by diminished action of the heart and general circulation, lessening the special sensibility of particular portions of the system, and exerting a happy, tranquilizing influence in cases of excessive reflex excitability.

V. *The Diseases in which its Use is Indicated.*—Its physiological effects are not so decided but that they may be easily modified or controlled by serious structural disease, or morbid processes going on in the system. The cases in which most benefit is derived from its internal use, are those in which functional derangement, rather than organic disease, is present. Its very beneficial effects in certain forms of insomnia have been noted by several writers, and I have to add my own observation in several marked cases, in which a nervous, wakeful condition was well controlled in persons where opium was not well borne, and with none of its unpleasant sequences. Acting upon the suggestion of Brown-Sequard, who has spoken very decidedly in its praise, Mr. Henry Behrend, an eminent English practitioner, commenced the use of bromide of potash in 1864, in cases of insomnia and restlessness, dependent upon nervous excitement and irritability. He cites several cases, where business men, by a severe overtaxing of their mental powers, were wholly unable to procure tranquil sleep. His prescription, in the instances cited, was 25 grs. of the bromide of potash three times a-day, dissolved in a little cold water, and with the happiest effects. Several of my associates have given me a like assurance of its beneficial influence as a sedative, and often very happily meeting the want where opium was not tolerated, and where valerian and other anti-spasmodics had failed.

Unhappily, the habits of our American people are such, that, for our overworked business men, for our overtasked children in schools, for our enervated females, who become morbidly sensitive mothers, a remedy like this seems especially needed, and it may be said in praise of this salt of potash, that, while it is a decided nervous sedative, it does not at all impair digestion, it does not constipate the bowels, and tends rather to allay than promote irritation of the urinary organs.

The dose for its sedative effect, in cases now alluded to, may vary from 15 grs. to 30, in solution, three times a-day. BROWN-SEQUARD assures us that he has given it in drachm doses, for several consecutive weeks, without any deleterious effect. As might be anticipated, it has already been very fully tried in *epilepsy*. Dr. WILKS, of Guy's Hospital, cites two cases, in which the exhibition of 5 grs. of the potash, three times a-day, was followed by recovery, when the ordinary remedies had failed.

His theory is this:—That in those cases there was local affection of bone or membrane, and that the remedy relieves by its absorbent power. I think it more probable, that the primary and favorable effect is upon the *nerve* tissue, diminishing its irritability, or else the iodide of potash, which is more potent as a stimulant to absorption, would act with superior energy and success, which is *not* the case. Dr. RODGERS, physician to West London Hospital, has recently tested its use in this disease, with very favorable results. He directs 15 to 20 grs. three times a-day, and has relieved, entirely, several cases that had baffled all previous effort. He dwells specially upon its *use* in epilepsy induced by excessive masturbation, one case, very severe in its development, yielding to a continued use of 25 grs. three times a-day, for several months.

A friend of mine related a case that had excited a good deal of interest in Boston recently, where physicians had failed to relieve a patient of most distressing pain in the cervical portion of the spinal cord, and which BROWN-SEQUARD controlled almost immediately by the administration of 20 grs. of bromide potash, administered three times a-day, and continuing its use during several weeks.

I have tested the use of bromide of potash, recently, in a case of tetanus, but without appreciable benefit; the case passing rapidly to a fatal termination, the exciting cause, whatever it may have been, brooking no restraint. Yet, in both these diseases, I am disposed to trust more to the use of bromine than to all other remedies.

One of its special effects is upon the nervous distribution to the upper portion of the respiratory tract. Hence, several papers have made their appearance upon its use in spasmodic affections of the larynx and bronchi. In spasmodic asthma, it has been spoken of with favor. In spasmodic croup, where there is a proclivity to its frequent return, I believe the tendency may be, in many instances, broken up by its judicious and timely use.

Whooping-cough is another affection, over which it exerts a very perceptible influence, and to its use in this disease, I invoke your careful consideration. Dr. GIBBS, of Westminster Hospital, London, reports a list of cases of this affection treated with bromide of ammonia. Not all were relieved. Those with spasmodic cough, however, were almost uniformly cured of the whoop; but, in proportion, as catarrhal symptoms were present, it failed to afford prompt relief. To infants, he administered from 3 to 5 grs. in solution three times a-day, increasing the dose to 4 and 8 grs. in the cases of older children

This drug is found also to produce a decided anæsthetic effect upon the genito-urinary passages. I have tested its ef fects upon irritation of the male urethra, and upon the genital irritation inducing nocturnal emissions, and have succeeded with this better than with any other remedy. A colleague cites a case of long-standing, habitual seminal emission, cured by the use of 20 grs. of bromide of potash three times a-day, its use being continued for a number of weeks.

Dr. DEBOUT, a French writer, cites a remarkable case of stricture of the urethra, so irritable that it was found impossible to dilate it in the ordinary manner, with bougies, and attended with excessive febrile excitement. He made use of a drachm, daily, of the bromide of potash, and succeeded beyond his most sanguine expectation; for, he says, as soon as the bromide had taken effect, the catheter could be introduced with greater and greater dimension, without any unfavorable effect. In this case, its hypnotic effect was very remarkable, for, although the patient had been almost sleepless for weeks previous, when only a half drachm had been administered, he obtained a good night's rest. In cases of irritability of the bladder, it has been equally successful. In some cases, its use has been discontinued from its excessive somnolent power, inducing too profound and prolonged sleep. Where there exists a morbidly sensitive condition of the mucous membrane of the genital organs, I think this remedy will be found to act very kindly, and, in the instances which have come under my notice, quite efficiently. It will be found serviceable in allaying the congestion and excitability of the genital organs of those habituated to masturbation, and may well be prescribed for its local sedative effect, during the period that the unfortunate victim is endeavoring to rid himself of the habit. I tried, in several cases, the use of bromide of potash in this morbid condition of the genital organs, where produced by nocturnal emissions, in doses of 3 to 5 grs. three times a-day, but failed of any satisfactory results till I had increased the dose to 15 and 20 grs. given at the same intervals. I anticipate that where there has been developed in these organs decided inflammatory action, the drug will, in a measure, fail to meet our desire; but in the case of decided local inflammation of the part, I still believe the exalted sensibility will be in a good degree controlled, and that this effect will favor recovery.

In all these cases, whether of epilepsy, tetanus, whooping-cough, bronchial irritability, or excitement of the genito-urinary organs, those cases are most favorably affected, where the patient is in a moderately sthenic condition; and, indeed, it will be found beneficial sometimes to sustain with decided tonic treatment at the same time that bromine is used for its tranquilizing effect. Several cases of extreme prostration and wakefulness were well relieved by the use of $\frac{1}{20}$ gr. of strychnine administered three times a-day, at mealtime, followed by 20 grs. of the bromide at bedtime. In all cases where it is administered to persons who are *enfeebled*, I think special care should be taken to support at the same time by appropriate tonics.

I pass, lastly, to consider its external use as a disinfectant and as an escharotic. The testimony of Surgeon WOODWARD, 22d Reg't Ill. Volunteers, to Surgeon GOLDSMITH, is very decisive, as to the effects of the vapor of bromine in wards devoted to the treatment of erysipelas. Others speak so decidedly in its praise when used in its volatile form to prevent the contagious effects of erysipelas, that I confess a desire to see their experiences more fully verified, and if the half is true which is asserted of it, we may well rank it next to chloroform—the second best of God's gifts to surgery. I am disposed fully to test it, by liberating a moderate amount of its vapor in all confined rooms where contagious or even pernicious diseases are to be treated, with the view of determining to what extent it has power to neutralize the *miasm* which is productive of contagion.

Finally, its use is recommended in the strongest terms by Surgeon GOLDSMITH, his hospital-assistants, and others, as an escharotic. The strong affinity which it has for the elements of the tissues causes it to act upon them in a similar manner to nitric acid; but it is found superior to the latter from the perfect neutralizing effect which it exerts upon the poison producing hospital gangrene. It has seemed, in many instances, an absolute specific against the ravages of this disease, controlling the progress of the sloughing, and changing the wound to the condition of a healthy ulcer in which, granulations springing up readily, the recovery was rapid. Of the manner in which it exerts its beneficial effect, both upon living and decomposing molecules, I am not able to speak; but the fact remains, that, when in its full strength, the solution of bromine has been applied to a gangrenous ulcer or wound, the peculiar *fetor* disappears, the rapid decomposition of tissue is arrested, the wound soon puts on favorable appearances, the patient demands food, improves in strength, and soon rallies from his critical condition. Of course there are numerous modifying influences which may prevent its favorable effects, but, so far as the local poison which developes and propagates hospital gangrene is concerned, it is asserted, upon reliable authority, to neutralize it promptly by a single thorough application, and that thereafter the wound is to be esteemed and treated as of *non*-malignant character. How fully the experience of the great body of army surgeons is confirmatory of the strong assurances of Surgeon GOLDSMITH upon this point, I am unable to say; but I close the paper at this point, to solicit from those who may have given it a full and impartial trial the result of their experience, as an external application in the prevention of erysipelas and in the cure of gangrene.

Two queries present themselves, which I will not withhold. The one is:—May not the presence of bromine with *iodine* in cod liver oil give to it an efficiency not possessed by any of its artificial imitations? The other:—May not bromine found in most of our saline and some of our chalybeate springs have given to them a more decided curative power than we have been wont to ascribe to medicinal springs?

With caution, that we do not anticipate too much from bromine as a curative agent, remembering that its action may be easily modified or entirely controlled by strong disturbing influences, as in acute disease, still I think it well for us to test, patiently and carefully, its effects upon the human economy; and I feel confident that in many instances, in the diseases before mentioned and perhaps others, we shall find in it a remedy which all must acknowledge to be essentially needed in the treatment of disease.

